# Predicting the Clinical Outcome of Triple-Negative Breast Cancer Based on the Gene Expression Characteristics of Necroptosis and Different Molecular Subtypes

**DOI:** 10.1155/2023/8427767

**Published:** 2023-02-20

**Authors:** Peng Luo, Zhaoqi Shi, Changshou He, Guojun Chen, Ji Feng, Linghua Zhu, Xiangyang Song

**Affiliations:** ^1^Department of General Surgery, Sir Run Run Shaw Hospital, Zhejiang University School of Medicine, Hangzhou 310020, China; ^2^Department of Oncology, HaploX Biotechnology, Shenzhen 518000, China; ^3^Department of Surgical Oncology, Sir Run Run Shaw Hospital, Zhejiang University School of Medicine, Hangzhou 310020, China

## Abstract

Necroptosis, a kind of programmed necrotic cell apoptosis, is the gatekeeper for the host to defend against the invasion of pathogens. It helps to regulate different biological processes regarding human cancer. Nevertheless, studies that determine the impact of death on triple-negative breast cancer (TNBC) are scarce. Therefore, this paper has comprehensively examined the expression as well as clinical significance of necroptosis in TNBC. ConsensusClusterPlus was used to establish a stable molecular classification that used the expression regarding the necroptosis-linked genes. The clinical and immune characteristics of different subclasses were evaluated. Then, the weighted gene coexpression network analysis (WGCNA) assisted in determining key modules, and we selected the genes exhibiting obvious association with necroptosis prognosis through the relationship with prognosis. The univariate Cox regression analysis together with least absolute shrinkage and selection operator (LASSO) techniques served for the construction of the necroptosis-related prognostic risk score (NPRS) model, and the pathway characteristics of NPRS model grouping were further studied. Finally, the NPRS, taking into account the clinicopathological features, used the decision tree model for enhancing the prognostic model as well as the survival prediction. First, two stable molecular subtypes with different prognosis and immune characteristics were identified using necroptosis marker genes. Then, the key modules were identified, and 10 genes significantly related to the prognosis of necroptosis were selected. Then, the clinical prognostic model of NPRS was developed considering the prognosis-linked necroptosis genes. Finally, the NPRS model, taking into account the clinicopathological features, adopted the decision tree model for enhancing the prognostic model as well as the survival prediction. Herein, two new molecular subgroups considering necroptosis-linked genes are proposed, and an NPRS model composed of 10 genes is developed, which maybe assist in the personalized treatment and clinical treatment guidance of TNBC patients.

## 1. Introduction

Breast cancer acts as a representative cancer type for females. Despite recent advancements in treatment, such as targeted therapy, hormone therapy, chemotherapy, and radiotherapy, breast cancer is still the primary cause of death related to cancer around the world [1]. High histological grade, proliferation rate, and ductal histology are characteristics of triple-negative breast cancer (TNBC). It is also linked to the deficient expression of the human epidermal growth factor receptor-2 (HER2), progesterone receptor, and estrogen receptor (ER) [2]. Approximately 10–20% of breast cancer patients have TNBC. Affected patients display high mortality and higher recurrence rates than those with any other breast cancer subtype [3]. The major systemic therapy for TNBC is routine chemotherapy. However, efforts to identify efficient targets to enhance clinical outcomes have been motivated by the dearth of molecular-focused based therapy and the dismal prognosis of TNBC patients.

Programmed necrosis, or necroptosis, is characterized by the permeability of the plasma membrane and modifications in the mitochondria, which cause the release of the cytoplasmic components into the extracellular environment and give rise to an inflammatory response [4]. Necroptosis refers to a death mechanism of necrotic cell independent of caspase. Its primary mediators through gene regulation are mixed-lineage kinase domain-like protein (MLKL), receptor-interacting protein kinase 1 (RIPK1), and RIPK3 [5]. There is mounting evidence that necroptosis is essential for controlling carcinogenesis and cancer progression. Najafov et al. noted that necroptosis can accelerate the metastasis of the tumors and T cell death [6]. By releasing the chemokine (C-X-C motif) ligand (CXCL), necroptosis in pancreatic cancer can encourage tumor cell motility and invasion [7]. The drug response of prostate cancer patients is influenced by necroptosis [4]. Necroptosis has been connected to antitumor immunity in cancer immunotherapy. According to Wang et al., the lncRNA SNHG1/miR-21-5p/TLR4 regulatory axis and the prognostic characteristics associated with necrosis were identified in gastric adenocarcinoma. According to Shen et al., the necroptosis signal is triggered in breast tissue cancer and is highly correlated with tumor growth and malignant tumor markers [8]. However, more information is required on the probable molecular mechanism of the TNBC and the exact function of necroptosis in the disease prognosis.

In this study, the mutational and immunological properties of stable molecular subgroups were identified by consensus clustering using genes relevant to necroptosis. Then, the WGCNA technique assisted in confirming the major modules, and such association was considered to select genes with close relation to necroptosis prognosis. The genes involved in necroptosis were discovered using univariate Cox regression analysis and the LASSO techniques. Then, a clinical prognostic model and risk model were developed, which could be useful in determining the prognosis of the TNBC patients and their particular treatment course.

## 2. Materials and Methods

### 2.1. Data Collection

All data regarding RNAseq, overall survival (OS) of the patients, and their TCGA-BRCA characteristics came from The Cancer Genome Atlas (TCGA) GDC API. The GSE103091 chip data set of the TNBC came from NCBI's Gene Expression Omnibus (GEO) database, which yielded 238 samples. Furthermore, the data set containing the TNBC sequencing data (METABRIC, Nature 2012 & Nat Commun 2016) was collected from the cBioPortal website (http://http://www.metabric.org/), hereinafter referred to as the cBioPortal data set. 74 necroptosis-linked genes used in an earlier study were also used in this report [9].

### 2.2. Data Preprocessing

Processing of TCGA-BRCA data set is as follows: (1) normal samples were discarded; (2) samples of TNBC were retained according to the clinical information table; (3) the samples without the birth time and OS status were eliminated, and the samples with a survival time < 10 years were retained. Processing of GSE103091 data set is as follows: (1) normal samples were discarded; (2) samples without their birth time and OS status were eliminated, and samples with an OS value of <10 years were retained; (3) the probe of chip data was converted into gene symbols; (4) the expression data of multiple genes corresponding to one probe were removed; (5) if a gene had multiple expression values, its average value was taken as the expression value. The following is the cBioPortal data set processing: (1) normal samples were removed; (2) samples of TNBC according to the clinical information table were retained; (3) the samples without the birth time and OS values were eliminated, and the samples with an OS value of <10 years were retained.

The TCGA and cBioPortal data sets (hereinafter referred to as RNAseq data sets) were combined, and the batch effect between different data sets was removed through the ComBat function of *sva* package [10].

### 2.3. Molecular Typing of the Necroptosis-Linked Genes

The ConsensusClusterPlus package served for consensus clustering for the building of a consistent matrix, thereby clustering and typing the samples [11]. We obtained samples' molecular subtypes from the expression data regarding genes related to necroptosis. The Pam algorithm and “maximum” served for measuring the distance, carrying out 500 bootstraps. 80% of patients were included in the training set in each bootstrap process. We defined the cluster number in the range of 2-10, meanwhile calculating the consistent matrix and the cumulative distribution function (CDF) for obtaining the molecular subtype, thereby assessing the optimal classification.

### 2.4. Construction of WGCNA

The WGCNA process was used for constructing a gene coexpression network [12]. First, for developing the gene expression similarity matrix, the following equation was used to estimate the absolute value regarding the Pearson correlation coefficient between the *i* and *j* genes: S_*ij*_ = |(1 + cor(*x*_*i*_ + *y*_*i*_))/2|. It has been noted that the coexpression network was in agreement with the scale-free network, that is to say, log (*k*) of a node with the connectivity, *k*, exhibited a negative link to log (*P* (*k*)) of the node probability, while the *R*^2^ value was >0.85. Thereafter, we converted the expression matrix to the adjacency matrix, which was then converted to the topological matrix. An average-linkage hierarchical clustering technique served for the clustering of genes related to the TOM. Based on the hybrid dynamic pruning tree, we defined a minimal no. of genes in every gene network module as 200. The eigengenes of each gene module were calculated after the dynamic cutting method had defined the gene modules. The modules were then clustered and analyzed, and the modules that were adjacent to one another were combined to form new modules. The Pearson correlation coefficient *b* of each pair of genes is represented by a soft threshold parameter, *β* [13]. This process further makes the strong relationship stronger, while making the weak relationship weaker at the index level: *a*_*ij*_ = |(1 + cor(*x*_*i*_ + *y*_*i*_))/2|^*β*^. The feature vector genes, or ME, serve as every module's representative genes and define the overall gene expression levels of the modules as follows: ME = princomp(*x*_*ij*_^*q*^) (*i* denotes the gene in the modulus *q* whereas *j* indicates the microarray samples in the modulus *q*). The expression pattern of the gene in all the data samples and the ME expression profile of a particular vector gene was utilized to identify the gene in a module using the Pearson correlation. This was described as the module membership (MM), where MM_i_^*q*^ = cor(*x*_*i*_, ME^*q*^), while ME denoted the expression profile of the gene *i*.

### 2.5. Prediction of Immunotherapy Reactivity

The tumor immune dysfunction and exclusion (TIDE) algorithm assisted in verifying the predictive influence exerted by the immune checkpoint inhibitor score (IMS) on clinical reactivity exhibited by immune checkpoint inhibitors (ICIs). TIDE algorithm is a calculation technique predicting the responsiveness of the immune checkpoint blockade (ICB), based on the gene expression profile [14]. It focused on assessing 3 cell types which restrict T cell infiltration (M2 subtype of cancer-associated fibroblast (CAF), tumor-associated macrophages (TAM), and myeloid-derived suppressor cells (MDSCs)) and 2 different mechanisms describing the tumor immune escape (the score on the dysfunction of the tumor infiltrating cytotoxic T lymphocytes (CTLs) and the score on the rejection of the immunosuppressive factors on CTLs).

### 2.6. Gene Set Enrichment Analysis (GSEA)

GSEA assisted in the pathway analysis for examining the pathways involving different biological activities in molecular subtypes. GSEA employed all the candidate gene sets in the Hallmark database [15].

### 2.7. Invasion Abundance Calculation of TME Cells

Microenvironment Cell Populations-Counter (MCP-Counter) served for analyzing the scores of the 10 immune cells [16], while the single sample gene set enrichment analysis (ssGSEA) analyzed the scores of the 28 immune cells [17]. Meanwhile, the Estimation of Stromal and Immune cells in Malignant Tumor tissues using Expression (ESTIMATE) process served for evaluating the general immune microenvironment infiltration score [18].

### 2.8. Constructing the NPRS Scoring System to Evaluate TNBC Samples

Identification of molecular subtype-related modules. Here, the entire expression profile of RNAseq was used for WGCNA analysis, and the most relevant modules of molecular subtypes were identified as the “key modules.” Then, the genes present in the key modules were extracted, wherein the genes showing a significant prognosis were chosen as those associated with the necroptosis phenotype. The total gene number was decreased using the LASSO regression [19] and stepAIC [20], while the prognosis-linked genes were acquired. For the construction of the NPRS scoring system, the following formula was employed for calculating the NPRS score of every patient: NPRS = *Σβ*i × Expi, where “Expi” represents necroptosis prognosis-associated genes' expressions and “*β*” represents the Cox regression coefficient regarding corresponding gene. A threshold value of “0” was taken into account for dividing patients into group with low NPRS risk and group with high NPRS risk. The Kaplan-Meier (KM) technique assisted in constructing the OS curve for the prognosis analysis, and a log-rank test helped to determine the significant differences between the groups.

### 2.9. Cancer Stem Cell

The expression data regarding pluripotent stem cell (PSC) samples (embryonic stem cell (ESC) as well as induced PSC (iPSC)) from Progenitor Cell Biology Consortium (PCBC) database were used for predicting as well as calculating the stem cell index by one-class logistic regression (OCLR) method. Firstly, only the sample data of ESC and iPSC are kept, which are collectively referred to as SC samples. The Ensembl IDs of SC samples are converted into gene symbol, and only the genes encoding proteins are kept. We obtain 78 SC samples at last, and the expression profiles regarding 8087 mRNA genes in each sample are kept. For the obtained expression profile, the average value assisted in centralizing each sample. Finally, the OCLR method in R package GelNet (V1.2.1) was used for calculating the weight vector of each gene on the processed data.

## 3. Results

### 3.1. Molecular Typing Based on the Necroptosis-Linked Genes

**Figure 1:**
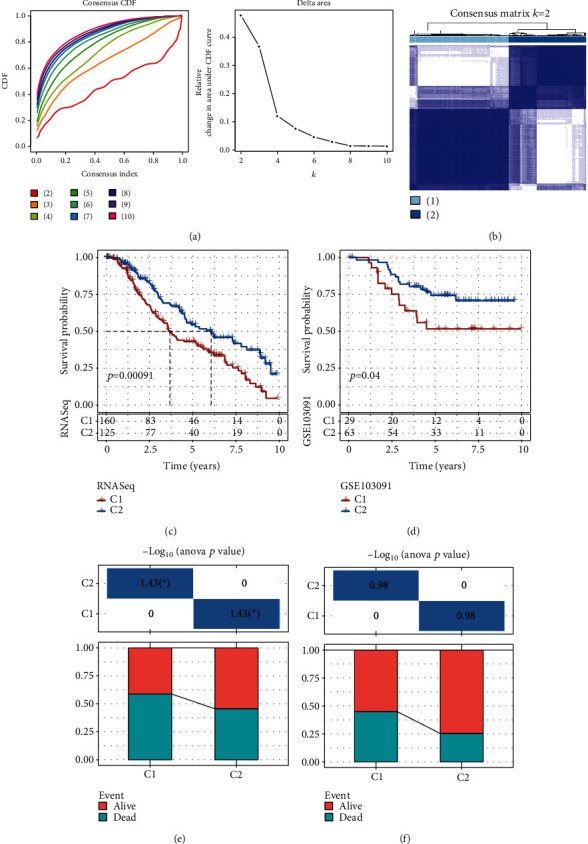
Consensus clustering analysis considering the prognosis of TNBC cell necroptosis-linked genes. (a) The CDF of different clustering methods for *k*-values ranging between 2 and 10. The relative change of the CDF AUC curve was determined for 2 to 10. (b) TNBC samples with clustering heatmap index *k* = 2 of RNAseq. (c) KM curve for the prognosis of necroptosis clusters in the RNAseq data set. (d) KM curve of OS between two clusters of necroptosis in GSE103091 cohort. (e) Subtypes' different survival status in the RNAseq data set. (f) Subtypes' different survival status in GSE103091 is different.

First, the expression exhibited by genes associated with necroptosis was retrieved from the RNAseq expression profile matrix. Eight genes were found to have significant OS values in the low and high gene expression groups (*p* < 0.01). The RNAseq data were then clustered using consensus clustering based on the eight necroptosis-linked genes related to prognosis, and the appropriate number of clusters was established using CDF. When cluster was chosen as 2, based on the CDF delta area curve, the clustering outcome presented a strong stability (Figures 1(a) and 1(b)). In the end, we selected *k* = 2 for generating two molecular subtypes. After examining the prognostic traits, the results showed that these two molecular subtypes had notable prognosis differences. Furthermore, the C1 group exhibited a poor prognosis, and C2 showed a better prognosis while the individuals with the C1 subtype had a significantly greater death rate relative to the C2 subtype (Figure 1(c)). Additionally, using the same technique to molecularly type the data from the GSE103091, it was clear that these two forms of molecular typing had significantly different prognoses, which was in line with the training set (Figure 1(d)). When the survival status of the various subtypes in the two data sets was further analyzed, it was discovered that C1 had a greater death rate than C2, which was aligned with the C1's poor prognosis (Figures 1(e) and 1(f)).

### 3.2. Immune Characteristics between Subtypes

**Figure 2:**
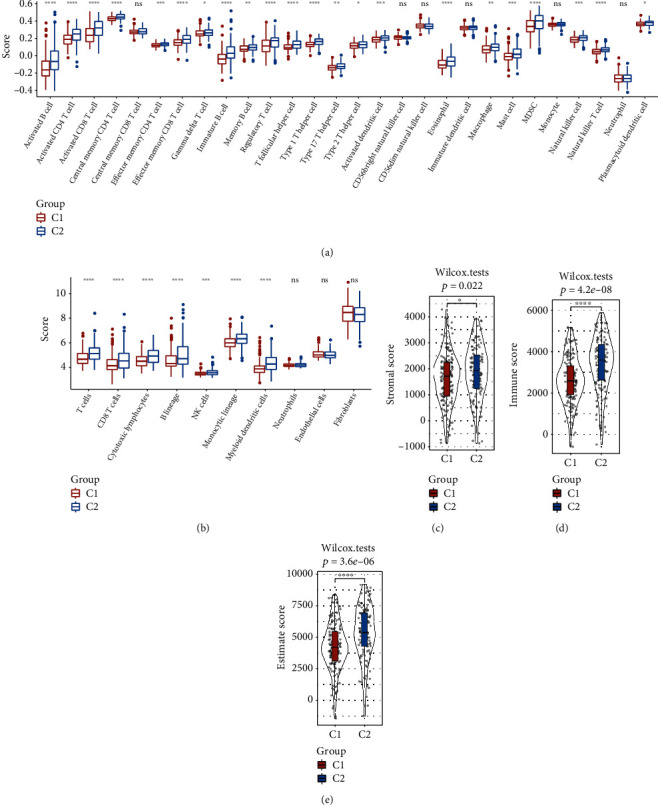
Immune-based properties of the 2 molecular subtypes in the RNAseq data set. (a) ssGSEA compared the 28 immune cell scores in the 2 subtypes. (b) MCP-Counter compared the 10 immune cell scores in the 2 subtypes. (c) ESTIMATE compared the StromalScore values in the 2 subtypes. (d) ESTIMATE compared the ImmuneScore values in the 2 subtypes. (e) ESTIMATE compared the ESTIMATEScore values in the 2 subtypes.

We compared the immunological features exhibited by the two subtypes for better revealing the immune microenvironment differences between both these subtypes. Significant differences were noted among 22 different types of immune cells by using the ssGSEA function of GSEA to analyze the scores of 28 types of immune cells. Compared to C1, C2 showed a higher immunological score (Figure 2(a)). The scores of the 10 immune cells were then analyzed using the MCP-Counter, and 8 of them showed different scores, while the C2 subtype also presented high scores (Figure 2(b)). The results of ESTIMATE analysis, which was used to assess the overall immune microenvironment infiltration score, were in agreement with those of MCP-Counter and ssGSEA (Figures 2(c)–2(e)).

### 3.3. Immunotherapy/Chemotherapy Differences between Subtypes

**Figure 3:**
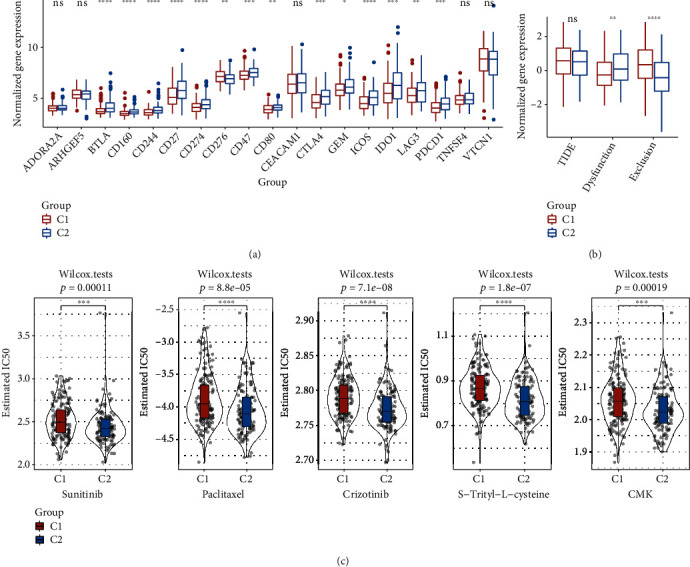
Immunotherapy/chemotherapy difference between the two subtypes in the RNAseq data set. (a) Immune checkpoints present differential expression between the various groups in the RNAseq data set. (b) The TIDE analysis difference between different groups in the RNAseq queue. (c) The box plots constructed using the calculated IC50 for sunitinib, paclitaxel, crizotinib, and bexarotene in RNAseq.

Furthermore, we analyzed the difference in immunotherapy between the two subtypes. Firstly, the section focused on comparing the subtypes in terms of different immune checkpoint expressions. Obviously, both the subtypes exhibited a differential expression of the 14 immune checkpoint genes (ICGs) (Figure 3(a)). As presented in Figure 3(b), the differences in subtypes of immunotherapy were analyzed. The TIDE scores of the RNAseq queues did not show obvious difference for both the molecular subtypes. C2 showed a higher dysfunction score than C1, while C1 showed a higher exclusion score than C2. In addition, the response degree of subtypes in the RNAseq cohort to traditional chemotherapy drugs (sunitinib, paclitaxel, crizotinib, S-trityl-L-cystine, and CMK) was also analyzed. The results indicated that C2 was more sensitive to these five drugs (Figure 3(c)).

### 3.4. WGCNA Analysis Identifying Molecular Subtype-Related Gene Modules

**Figure 4:**
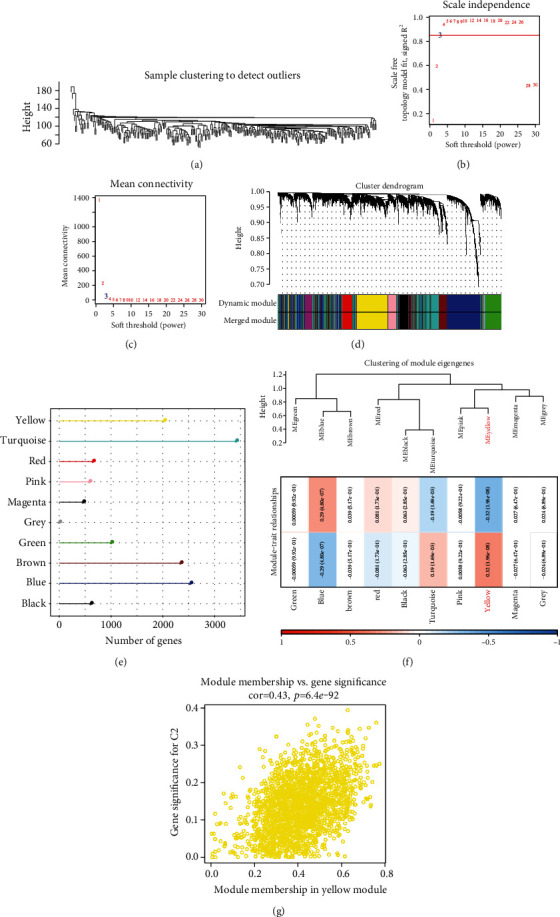
The hub gene in the RNAseq data set was identified by WGCNA. (a) Clustering trees of every sample. (b) Scale-free exponential analysis on soft threshold powers (STP, *β*). (c) The average connectivity between different STPs was analyzed. (d) The dendrogram was constructed using DEGs based on different degrees of measurement clustering (1-TOM). The different colored bands indicated the results of automatic block analysis. (e) Statistics of gene no. in each module. (f) The relationship between the module feature vector of every module and clinical data. (g) Scatter diagram that plots the MM vs. GS values for C1 in the yellow module.

RNAseq data set served for identifying molecular subtype-associated gene modules using the “WGCNA” R software package. First, we clustered the samples and selected the coexpression module (Figure 4(a)). For ensuring the network to be scale-free, we selected a *β* = 3 value (Figures 4(b) and 4(c)). In addition, the following settings were used to merge similar clusters into the new modules, i.e., deepSplit = 2, height = 0.25, and minModuleSize = 200. This yielded 10 modules in total (Figure 4(d)). It is worth noting that the grey module represented the gene set incapable of being aggregated with the other modules.  Figure 4(e) highlights the gene statistics regarding every module. Further, the relationship between every module and the molecular subtype was analyzed, and obviously, the yellow module showed an obviously positive relationship with the C1 subtype and a significantly negative relationship with the C2 subtype (Figure 4(f)). The module membership (MM) and the gene significance (GS) of the genes included in the yellow module were significantly and positively related (*r* = 0.43, *p* < 1e − 5) (Figure 4(g)). Through the function enrichment of genes in the yellow module based on the “ClusterProfiler” R software package, the top 10 pathways with the most significant enrichment were shown. The yellow module presented obvious enrichment in pathways like the neurotrophin signaling pathway, Huntington's disease, and sphingolipid signaling pathway (Supplementary Figure S1). Finally, the yellow module which was highly related to molecular typing was identified as the key gene module related to molecular typing.

### 3.5. Determination of Genes Related to Necroptosis Phenotype

**Figure 5:**
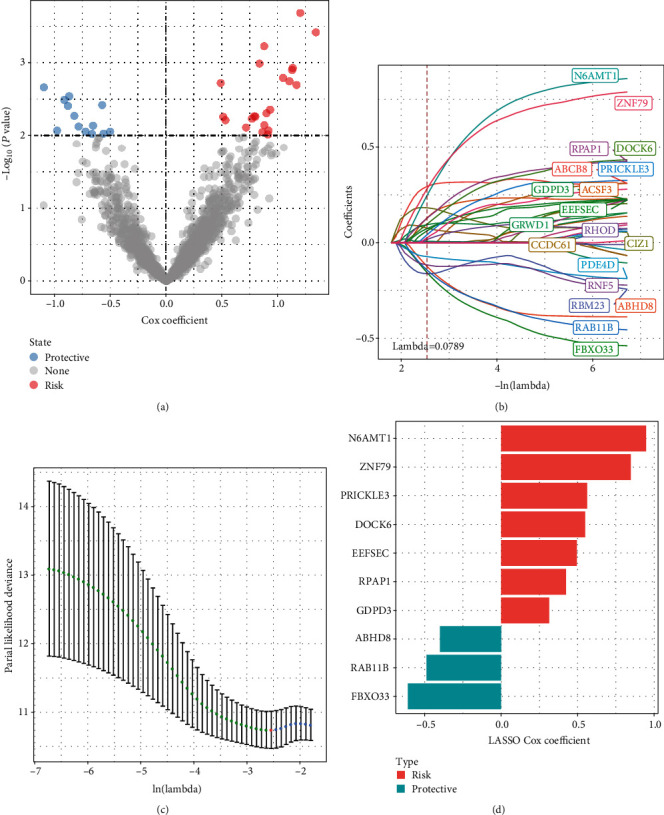
Identifying the genes related to necroptosis in the RNAseq data set. (a) Univariate regression between DEGs and TNBC prognosis. (b) LASSO coefficient distribution regarding genes possessing prognostic value. (c) A 5 cross-validation was utilized for selecting the optimal parameters in the model. (d) The coefficient of each gene in the optimal model.

The RNAseq data were then divided by 7 : 3 with 0.7 being the proportion of the training data set for the yellow module genes that showed a strong correlation to the molecular subtypes determined by WGCNA in the previous step. The training data set that included these genes underwent univariate Cox regression analysis, finding 35 genes with a greater influence on prognosis (*p* < 0.01). It had 13 “protective” genes and 22 “risk” genes (Figure 5(a)). Additionally, the 35 genes in the RNAseq data set were compressed using LASSO regression for lowering the gene no. in the risk model. First, we focused on examining the change trajectory presented by each independent variable. The no. of independent variable coefficients being close to 0 elevated as the value of lambda gradually increased (Figure 5(b)). We adopted10-fold cross-validation for constructing the model, meanwhile examining the confidence interval (CI) for each lambda (Figure 5(c)). The model was optimized at lambda = 0.0789. Hence, we selected 20 genes as the target genes for the subsequent analyses when lambda = 0.0789. Stepwise multivariate regression analysis and the Akaike information criteria (AIC) served for optimizing the model combining the results of the LASSO analysis of the 20 genes. Finally, 10 genes were identified as necroptosis-linked genes from LASSO analysis that affect prognosis (N6AMT1, ZNF79, PRICKLE3, DOCK6, EEFSEC, RPAP1, GDPD3, ABHD8, RAB11B, and FBXO33) (Figure 5(d)).

### 3.6. Clinical Prognosis Model Establishment and Validation

**Figure 6:**
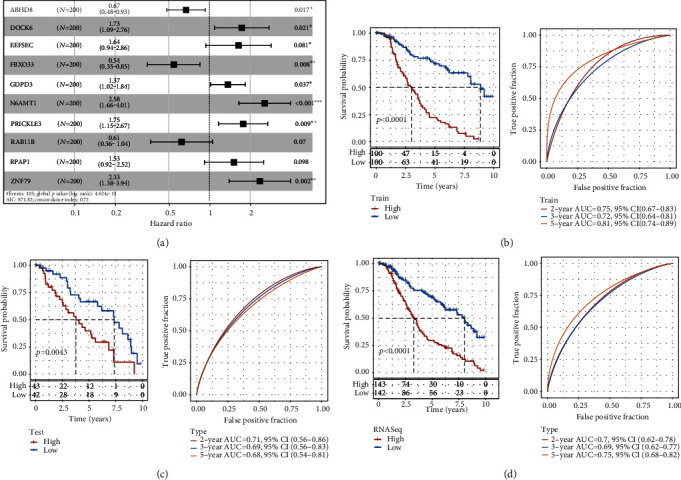
Clinical prognostic model development and examination. (a) Multivariate Cox regression-based forest map of model genes in RNAseq data set. (b) ROC and the KM survival curves depicting NPRS in RNAseq training queue. (c) ROC and KM survival curves depicting NPRS in RNAseq validation queue. (d) ROC and KM survival curves depicting NPRS in RNAseq queue.

The risk coefficients of 10 genes were obtained (Figure 6(a)). Then, according to the formula defined by the score of necroptosis of our samples, the necroptosis-linked prognostic risk score (NPRS) of every sample was determined. In the RNAseq training, the RNAseq validation, and the entire RNAseq data sets, the RS of each sample was calculated, respectively, and the best cutoff was categorized into group with high risk and group with low risk, followed by building their KM and ROC curves, respectively. The results of the RNAseq training, RNAseq validation, and the entire RNAseq data sets revealed the poor prognosis of high NPRS and good prognosis of low NPRS, and the ROC curve had a high AUC (Figures 6(b)–6(d)). This result had also been verified in TCGA data sets, GSE103091, and cBioPortal (Supplementary Figure S2 A-C).

### 3.7. Pathway Characteristics between NPRS Groups

**Figure 7:**
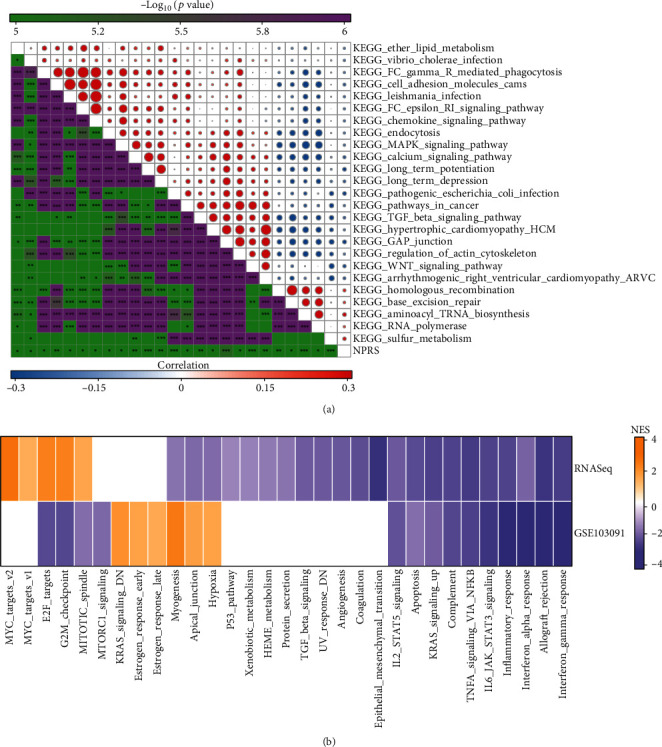
Pathway difference analysis of NPRS groups in RNAseq queue. (a) The results of correlation analysis between KEGG pathway and NPRS whose correlation with NPRS is greater than 0.2. (b) Heatmap depicting the normalized enrichment scores (NESs) of the Hallmark pathways that were estimated after comparing the NPRS-high with the NPRS-low (at the false discovery rate (FDR) < 0.05). ns: no significance. ^∗^*p* < 0.05, ^∗∗^*p* < 0.01, ^∗∗∗^*p* < 0.001, and ^∗∗∗∗^*p* < 0.0001.

The section focused on analyzing the correlation between the biological functions and NPRS of different samples in the RNAseq data set. Accordingly, these pathways were negatively correlated with NPRS of samples, and these pathways were mainly tumor-related (KEGG_WNT_SIGNALING_PATHWAY, KEGG_TGF_BETA_ SIGNALING_PATHWAY) (Figure 7(a)). Next, we selected some important biological pathways to check for activation in different NPRS groups. It was noted that in the RNAseq queue, compared with NPRS-low, 5 pathways were activated and 22 pathways were inhibited in NPRS-high, and 6 pathways were activated and 14 pathways were inhibited in the GSE queue (Figure 7(b)). On the whole, KRAS signaling up and TNF-*α* signaling via NF*κ*B, etc., were the main inhibited pathway in the NPRS-high group. Besides, we calculated the mRNAsi in high group and low data set and found that high group had higher mRNAsi (Figure S3).

### 3.8. NPRS in Combination with Clinicopathological Features for Enhancing the Prognostic Model and the Survival Prediction

**Figure 8:**
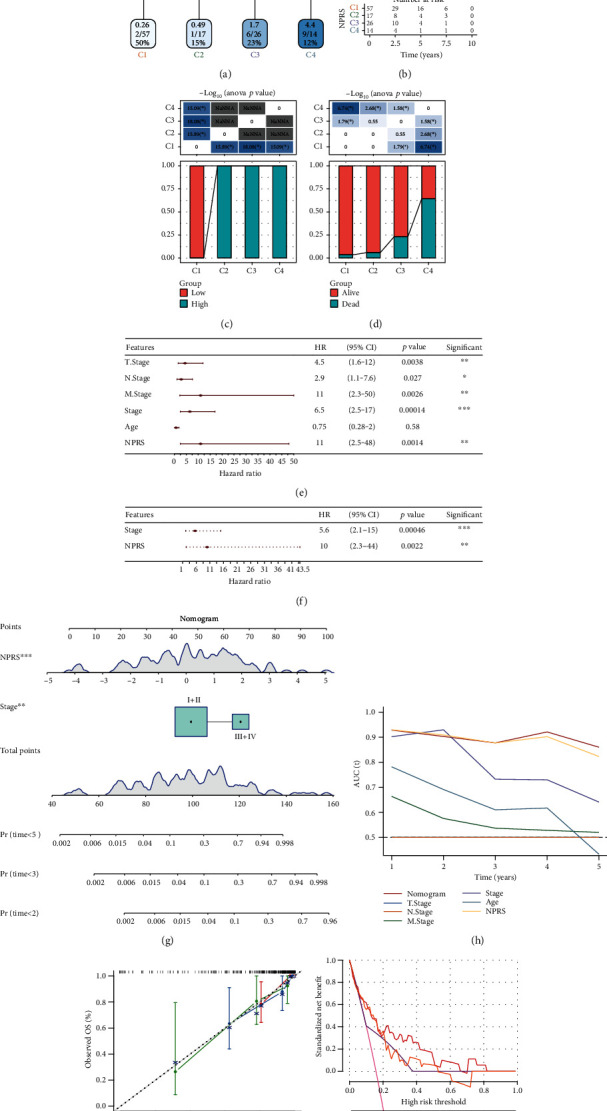
Nomogram of prognostic risk model in RNAseq data set combined with the clinical and pathological characteristics. (a) Patients with the full-scale annotations (RS, gender, age, and TNM Stage) were employed for constructing the survival decision tree and optimizing the risk stratification. (b) Significant differences noted in OS values noted in the 4 risk subgroups. (c, d) A comparative analysis between groups. (e, f) Univariate and multivariate Cox regression analyses of the RS and clinicopathological traits. (g) In comparison to the different clinicopathological features, the nomogram showed the highest capacity for OS prediction. (h) Nomograph model. (i) Calibration curve regarding nomograph in 2, 3, and 5 years. (j) Decision curve regarding nomograph. ns: no significance. ^∗^*p* < 0.05, ^∗∗^*p* < 0.01, ^∗∗∗^*p* < 0.001, and ^∗∗∗∗^*p* < 0.0001.

The age, N stage, T stage, M stage, and the NPRS of patients with TNBC in the RNAseq cohort were considered for constructing the decision tree. As found, only NPRS, stage, and age could be still observed in the decision tree, and four different risk subgroups (i.e., C1, C2, C3, and C4) were determined (Figure 8(a)). Significant differences were noted in the OS values of the four risk subgroups, and the prognosis of the C4 subgroup was the worst (Figure 8(b)). We analyzed the distribution of the four risk subgroups, finding the risk subgroups C2, C3, and C4 included the NPRS-high patients, while the patients in the C1 group were NPRS-low patients (Figure 8(c)). In addition, some differences were also noted in the OS status of the patients in various risk subgroups, and the distribution of death cases in the C4 subgroup was the highest (Figure 8(d)). As revealed by the univariate and multivariate Cox regression analyses of the NPRS and clinicopathological traits, the NPRS was a prognostic factor with the largest significance (Figures 8(e) and 8(f)). As revealed by the univariate Cox regression analysis results, NPRS could result in poor survival rate of the patients (*p* = 0.0014, HR = 11, 95% CI: 2.5-48) and remarkably predict the poor OS (Figure 8(e)). The multivariate Cox regression analysis adopted the statistically significant characteristic factors in the univariate Cox regression, finding NPRS as an independent risk factor (*p* = 0.0022, HR = 10, 95% CI: 2.2-44) (Figure 8(f)). For quantifying the risk evaluation and the survival probability regarding TNBC patients, we integrated NPRS with other clinicopathological characteristics to establish a nomogram, as shown in Figure 8(g). From the model results, NPRS had the highest effect on the OS prediction. Further, the calibration curve served for assessing the prediction accuracy of model. Obviously, the 3 calibration points in 2, 3, and 5 years presented similar prediction calibration curve to standard graph (Figure 8(g)), which suggested that the nomogram displayed an effective prediction performance. Nomogram and NPRS had higher AUC (Figure 8(h)). Additionally, the decision curve analysis (DCA) served for assessing the model reliability. The nomograms also showed a significantly higher advantage than the other curves (Figure 8(i)). Compared with other single clinicopathological features, nomograms showed the strongest ability to predict survival (Figure 8(j)).

## 4. Discussion

Recently, the necroptosis signaling pathway has been considered as a significant event in the regulation of tumorigenesis and progression, which remarkably impacts tumor development, tumor necrosis, tumor metastasis, and tumor immune response, suggesting the potential of targeting necroptosis as a new tumor treatment [21]. It is reported that the current correlation between necroptosis and antitumor immunity is summarized: there are two strategies to trigger antitumor immunity through necroptosis: (1) inoculation of necrotic tumor cell vaccine: damage-associated molecular patterns (DAMPs) were released by necrotizing apoptotic tumor cells to promote the maturation of bone marrow-derived dendritic cells (BMDCs), cross excitation of the effector T cells, and the resulting cytotoxic effects. A higher concentration of interferon-*γ* was noted in this process, which may represent a different anticancer method used by the CD8^+^ T cells. (2) Inoculation of necrotic fibroblast vaccine: necrotic cells secrete the NF-*κ*B-derived signals, which leads to DC cell activation, increases the antigen load of normal tumor cells, and increases the CD8^+^ T cell-mediated tumor control [22].

TNBC is mostly seen in young women. Its clinical course is invasive, the probability of visceral metastasis and brain metastasis is high, and it shows a worse prognosis compared to other forms of BC. In addition, the efficacy of endocrine therapy for hormone receptors and targeted therapy for blocking HER2 is poor. Chemotherapy is only the main treatment, but it is very susceptible to drug resistance, which seriously affects the prognosis. Immunotherapy can prolong the survival of patients. Potential biomarkers of TNBC immunotherapy response include high tumor mutation burden (TMB), TILs, and immune infiltration transcriptional characteristics [23]. In this study, two molecular subtypes (i.e., C1 and C2) of TNBC were defined by the expression profile of necroptosis-related genes. Significant prognostic differences were noted between the 2 molecular subtypes, and C1 showed a poor prognosis. The interplay between the many cell types that make up the TME is connected to carcinogenesis, tumor progression, treatment resistance, and immune infiltration matrix [24]. We determined the immune cell infiltration level in the RNAseq cohort of patients, finding higher immunological score of the C2 subtype relative to the C1 subtype. Cancer immunotherapy depending on the ICIs has shown great clinical success in recent years. The link between various antitumor immunity and cell death mechanisms has been recently discovered. Induced pyroptosis, necroptosis, and ferroptosis with ICI exhibit a synergistic increased antitumor effect [22]. After investigating the response of the subtypes to immunotherapy, most ICGs presented differential expression in the 2 molecular subtypes. Considering immunotherapy scores, the C2 group had a higher dysfunction score than the exclusion score of the C1 group. In addition, the C2 subtype was more sensitive to sunitinib, paclitaxel, crizotinib, S-trityl-L-cystine, and CMK. These findings suggest that patients with the C1 subtype who have poor immune cell infiltration should consider alternative treatment strategies because they have a bad prognosis and meanwhile show a poor response to pharmacological therapy.

Furthermore, LASSO regression and AIC algorithm were used to obtain the prognostic risk model constructed by 10 genes (N6AMT1, ZNF79, PRICLE3, DOCK6, EEFSEC, RPAP1, GDPD3, ABHD8, RAB11B, and FBXO33). DNA N6-methyladenine (6 mA) is seen to be a novel form of DNA methylation that is present in different eukaryotic cells. Studies have found that overexpression of methylase N6AMT1 increases the viability of hepatocellular carcinoma (HCC) cells, inhibits apoptosis, and enhances migration and invasion [25]. A different study found the N6AMT1 showed an obviously lower expression in the TNBC tissues relative to the normal tissues [26]. It was found that DOCK6 presented overexpression in the gastric cancer tissues, and the positive expression is related to gastric cancer metastasis, indicating that the gastric cancer patients showed a poor prognosis. Through miR-148b-3p, Rac1 and Cdc42 can be activated, which directly affect the motility of gastric cancer cells, and can serve as a new treatment strategy specific to gastric cancer [27]. EEFSEC presents an obvious upregulation in prostate cancer cells, and the high expression leads to poor prognosis, affecting the proliferation, migration, invasion, and cell cycle of 22Rv1 cells [28]. Naka et al. found that the deficiency of GDPD3 reduced the levels of some lysophosphatidic acid (LPA) and lipid mediators in chronic myeloid leukemia cells. Its deficiency also activated AKT/mTORC1 signaling pathway and cell cycle and inhibited the interaction of Foxo3a/*β*-catenin in the stem cell nucleus of chronic myeloid leukemia (PMID: 32943626). RAB11B-AS1 is a natural lncRNA upregulated in human BC, which enhances the BC cell in terms of the invasion and migration and promotes tumor angiogenesis and distant metastasis of breast cancer [29]. There are no relevant reports of ZNF79, PRICKLE3, RPAP1, ABHD8, and FBXO33 in cancer, and more research is still needed.

Recent research has demonstrated that during tumor formation, tumor necroptosis might be induced by death factors to engage in the RIPK1 and RIPK3 pathways [30]. A new study noted that RIPK1 is necessary for inflammatory disorders instead of for the growth or metastasis of tumors [31]. As a result, the fundamental mechanism behind tumor necroptosis during carcinogenesis is yet unknown. According to research by Baik et al., Z-DNA binding protein 1 (ZBP1), rather than RIPK1, is the primary regulator of tumor necroptosis in tumor formation in breast cancer. ZBP1 deletion prevents tumor cells from necroptosis during tumor formation, preventing tumor metastasis in the MVT-1 BC model. For determining the correlation between NPRS and biological functions, different tumor-related pathways (KEGG_WNT_SIGNALING_PATHWAY, KEGG_TGF_BETA_SIGNALING_PATHWAY) were negatively correlated with NPRS of the samples through ssGSEA analysis. Next, it was seen that the different NPRS groups activated different pathways. The results showed that in the RNAseq queue, compared with NPRS-low, 5 pathways were activated and 22 pathways were inhibited in the NPRS-high queue, and 6 pathways were activated and 14 pathways were inhibited in the GSE queue. On the whole, KRAS signaling up, TNF-*α* signaling via NF-*κ*B, etc. are the main inhibited pathways in the NPRS-high group. KRAS signaling is associated with cancer progression in many cancers. A few recent studies indicated that the KRAS mutations are found in about 30% of TNBC patients [32]. Through cell-mediated apoptosis and immune processes, tumor necrosis factor-*α* (TNF-*α*) was a complex connector between inflammation and cancer. It has been stated that TNF-*α* is involved in cell proliferation, tumor migration, tumor metastasis, matrix degradation, invasion, and angiogenesis [33]. Many researchers who investigated the molecular mechanism of necroptosis highlighted the role played by the TNF signaling pathway. Usually, TNF activates proinflammatory genes through NF-*κ*B signaling to induce an inflammatory response and activate downstream NF-*κ*B and MAPK pathways, resulting in increased proinflammatory gene expression [34]. Therefore, necroptosis exhibits an obvious association with tumorigenesis and development, and its internal complex regulatory mechanism remains to be further studied.

Future researches shall be conducted to combine the fundamental experiments and be deepened from the functional perspective. The study failed to consider other factors due to the lack of basic data on clinical follow-up, especially the diagnostic details.

Two TNBC necroptosis-related subtypes were found in this investigation, and subtype verification and molecular identification were carried out using separate data sets. However, this study has some limitations, though different molecular subtypes were investigated and confirmed in two different data sets. These findings are based on retrospective research, so additional functional studies are required to investigate the molecular roles played by the biomarkers in the 2 molecular subtypes. To further elucidate the mechanism of these targets, it is also required for the validation by using tissue samples and clinical patients. Overall, this research offers several prospective biological targets for developing fresh immunotherapies, which might eventually support the individualized treatment of TNBC patients.

## 5. Conclusions

The prognosis of TNBC was accurately predicted using a new prognostic risk model that included 10 genes linked to necroptosis. Further investigation shall be conducted for fully understanding the intricate molecular functions of these 10 genes. This study further highlighted the connection between necroptosis-linked genes and TNBC prognosis and immunotherapy. The findings of this study might enable clinical TNBC patients to receive precise and individualized care.

## Data Availability

The data set analyzed in this study could be found in GSE103091 at https://www.ncbi.nlm.nih.gov/geo/query/acc.cgi?acc=GSE103091.

## Supplementary Materials

Supplementary 1 Figure S1: functional enrichment analysis on genes in the yellow module.

Supplementary 2 Figure S2: clinical prognostic model development and examination. (A) ROC curves and KM survival curves of NPRS in TCGA cohort. (B) ROC curves and KM survival curves of NPRS in GSE103091 queue. (C) ROC curves and KM survival curves of NPRS in cBioPortal queue.

Supplementary 3 Figure S3: high group presented higher mRNAsi relative to low group.
