# Supplementary figures and images for: Predicting the Clinical Outcome of Triple-Negative Breast Cancer Based on the Gene Expression Characteristics of Necroptosis and Different Molecular Subtypes
Source: Stem Cells Int. 2023 Feb 20;2023:8427767. doi: 10.1155/2023/8427767 (PMC10234373; doi:10.1155/2023/8427767)

**A**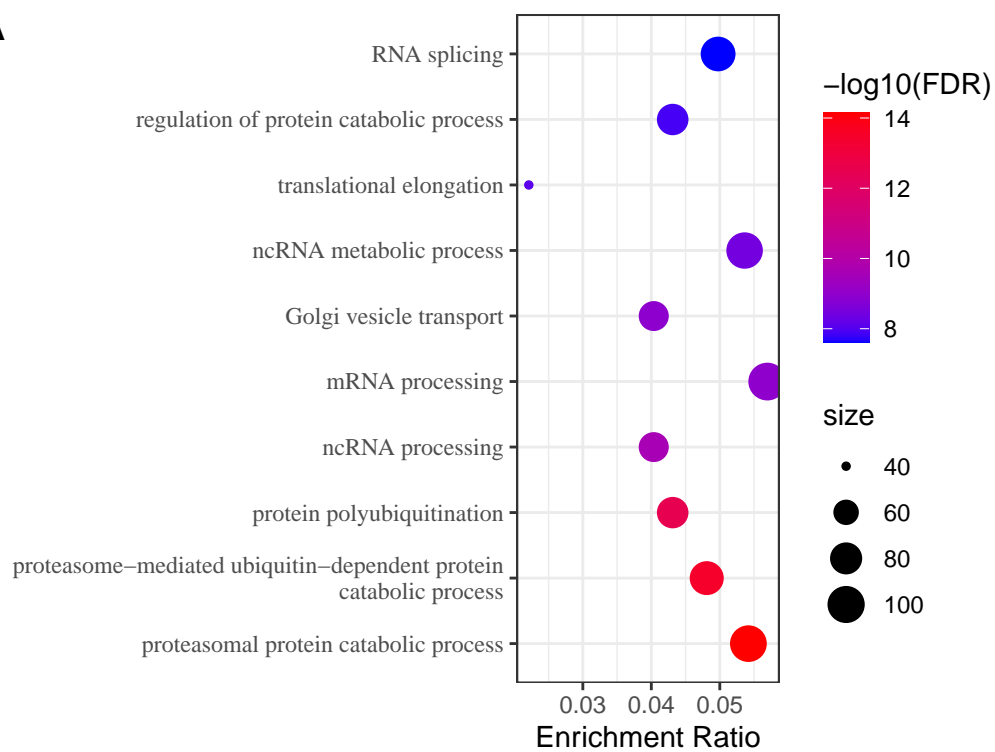**B**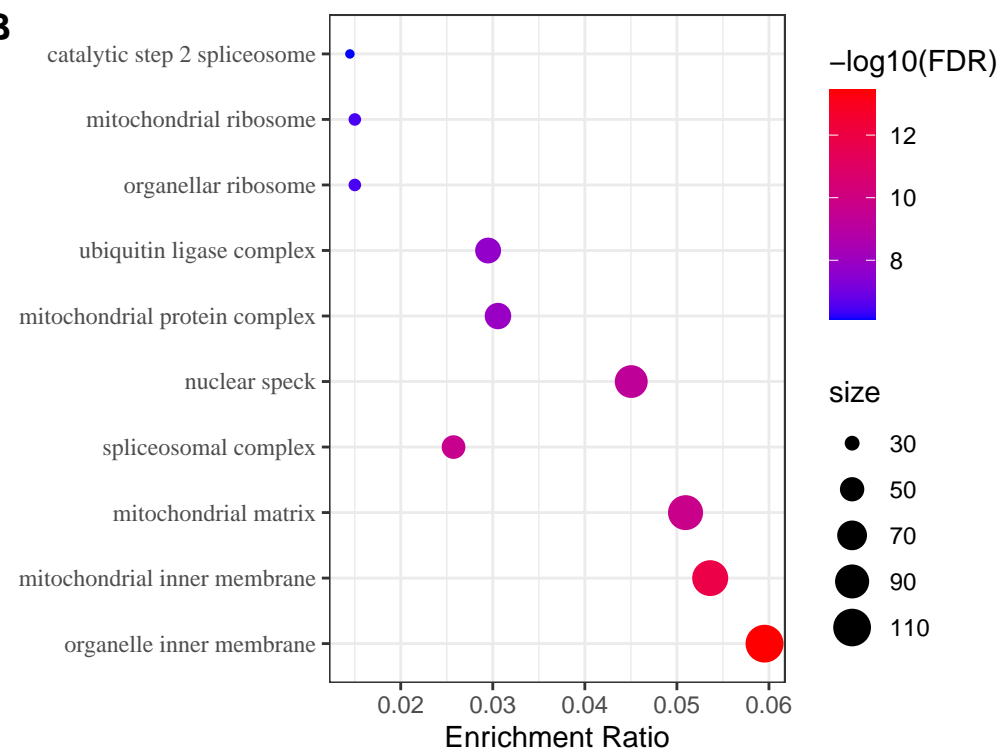**C**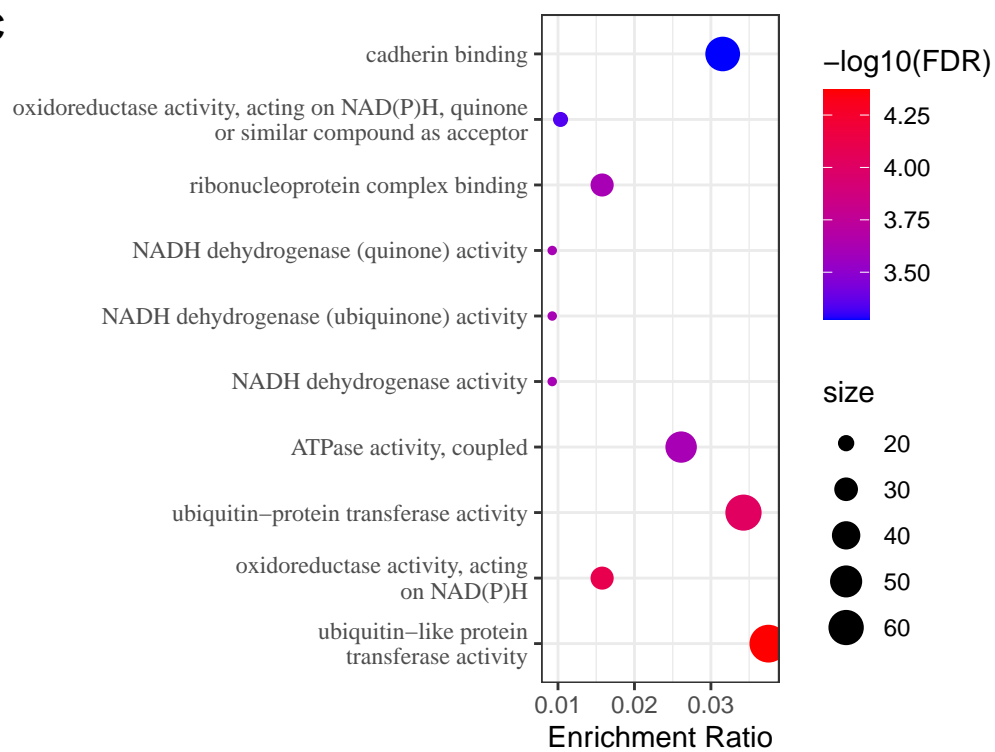**D**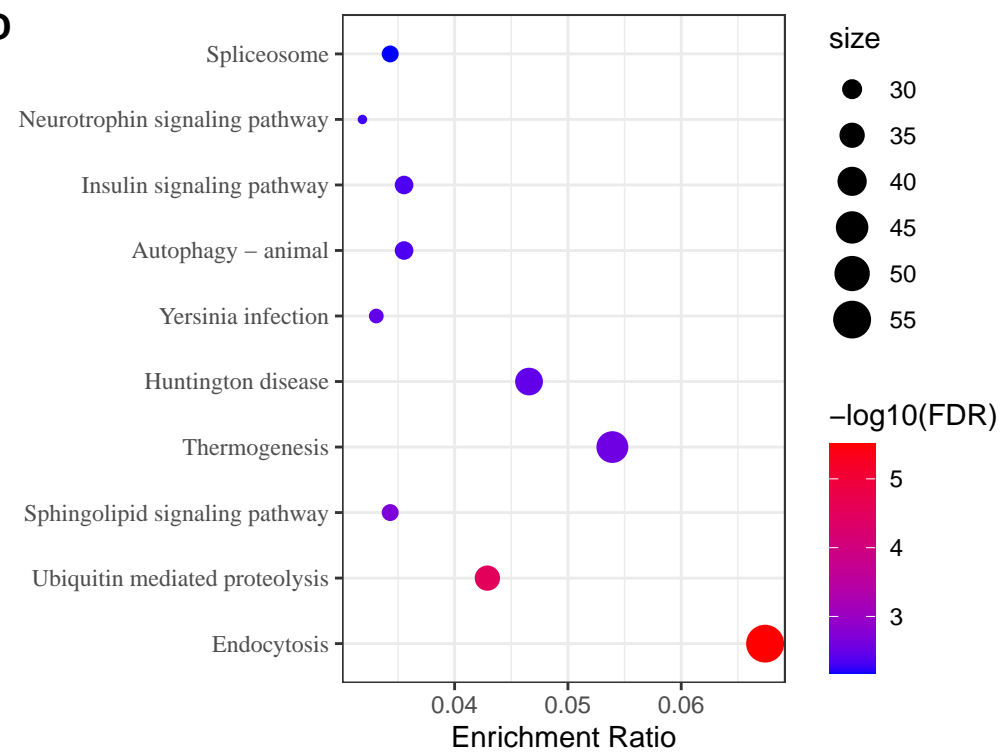

Supplement: Supplementary 1 — Figure S1: functional enrichment analysis on genes in the yellow module. [file 8427767.f1.pdf]

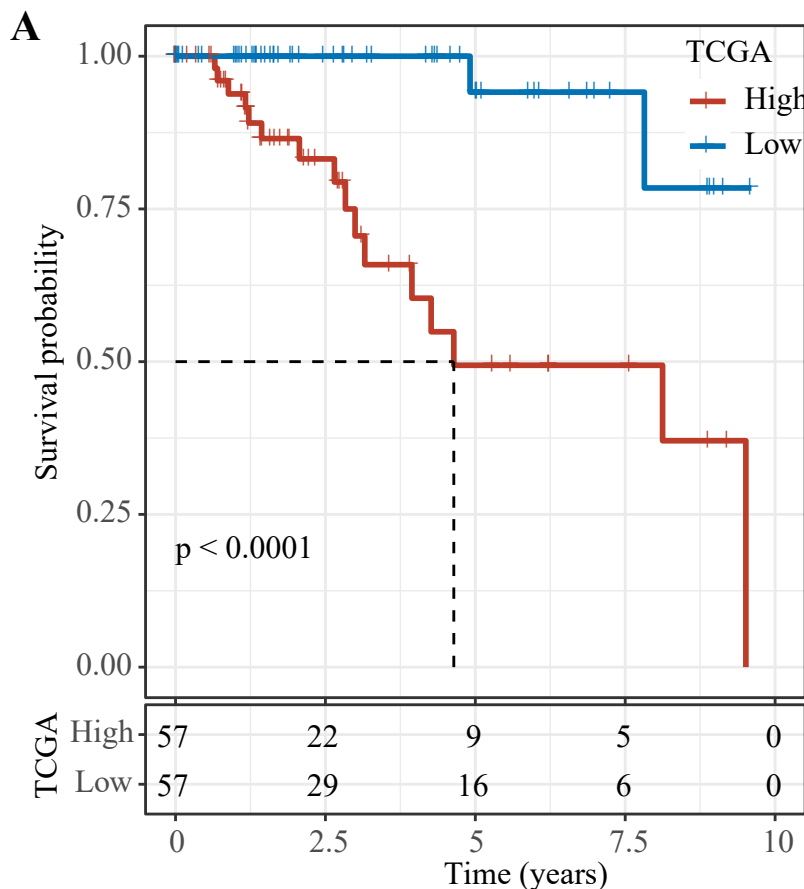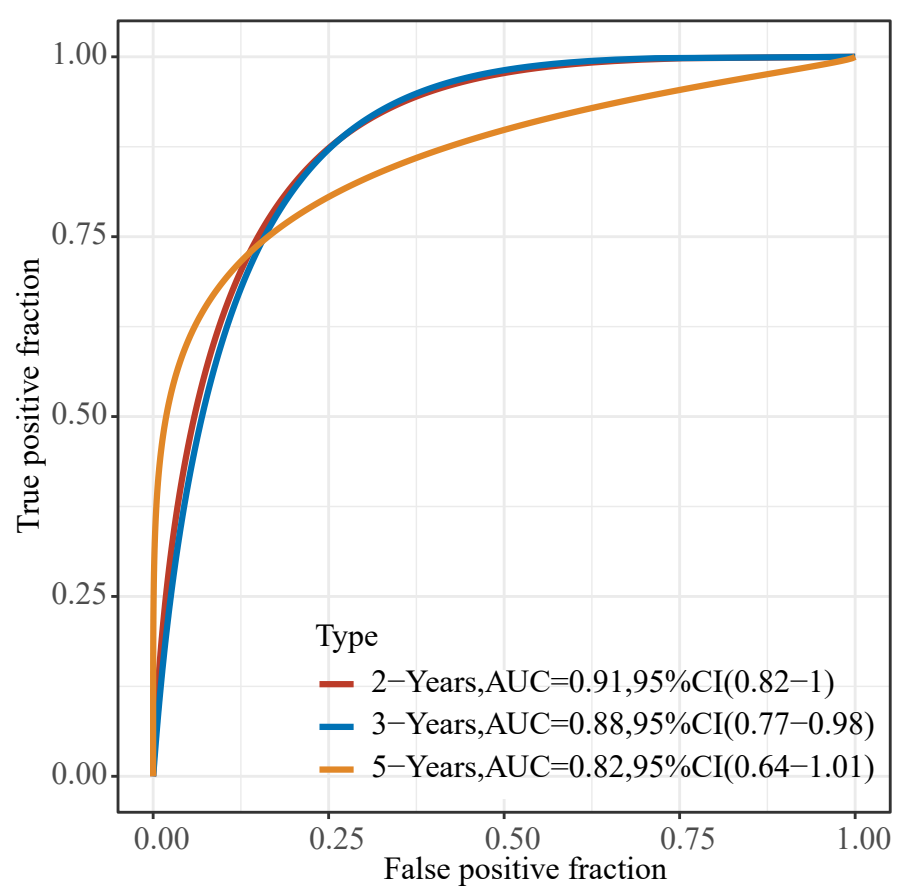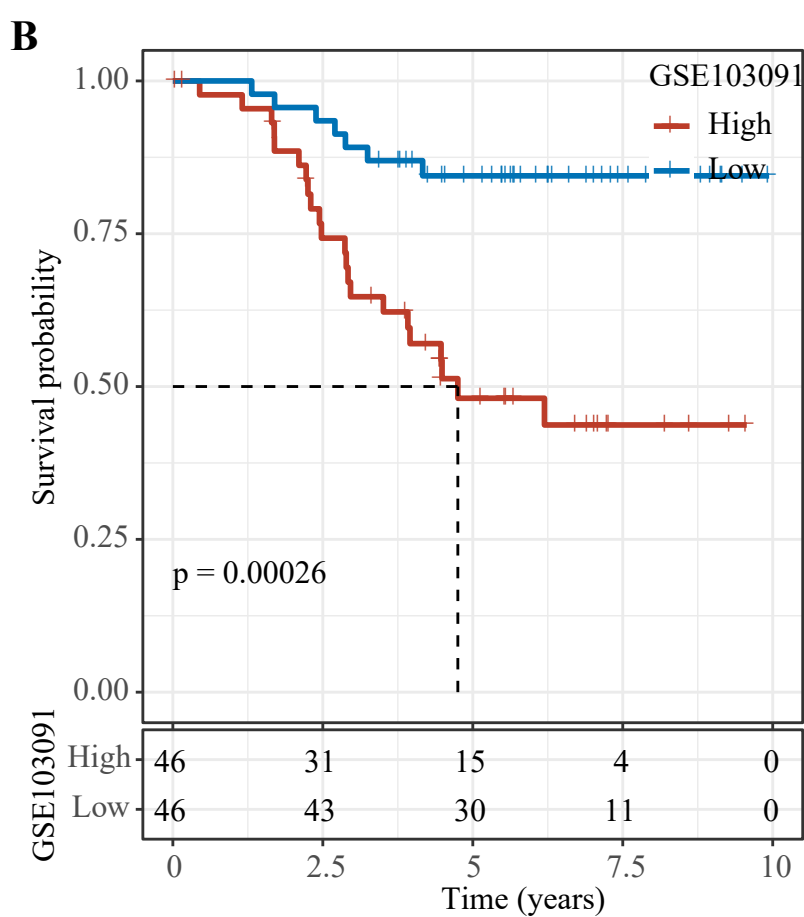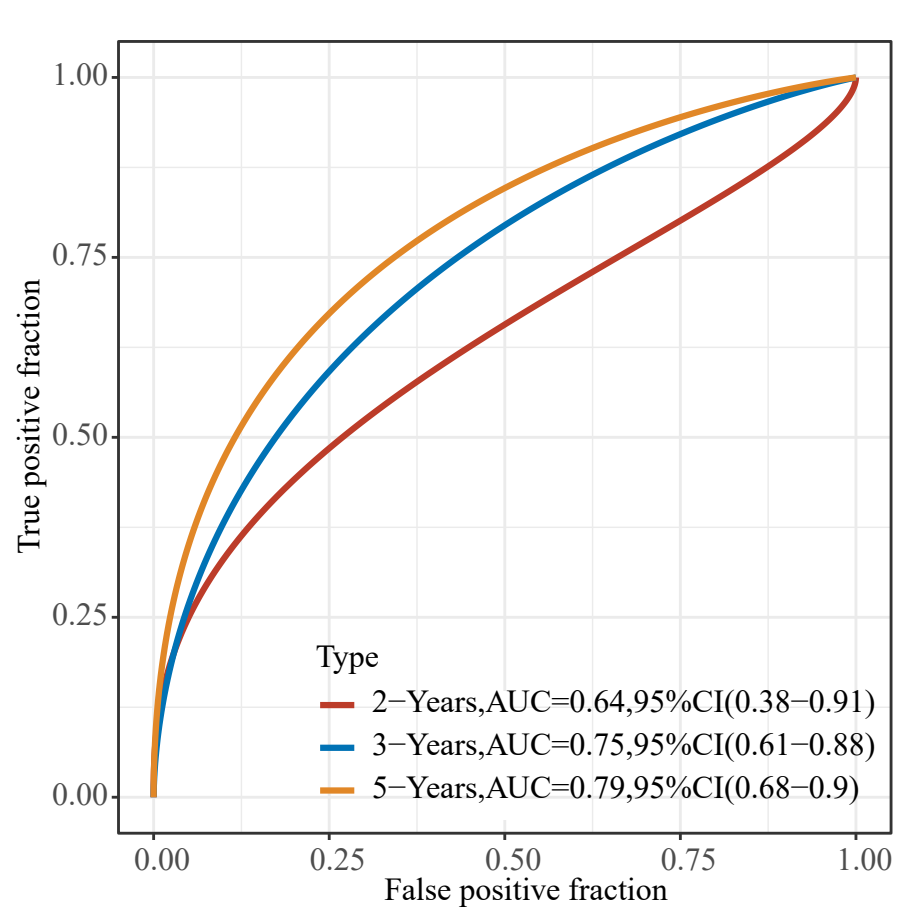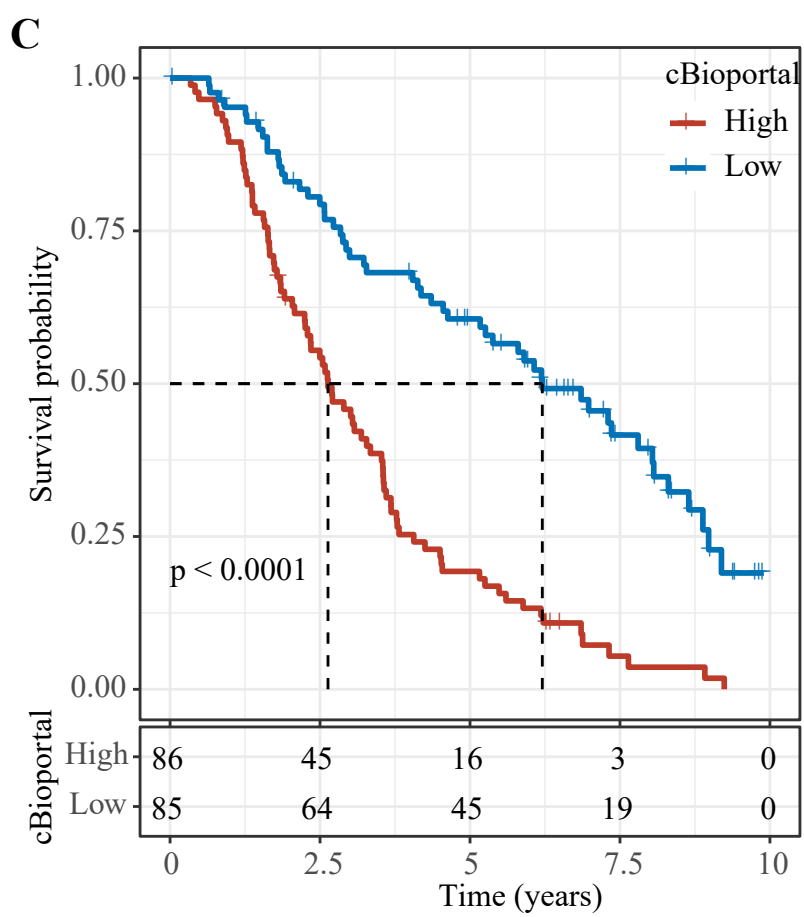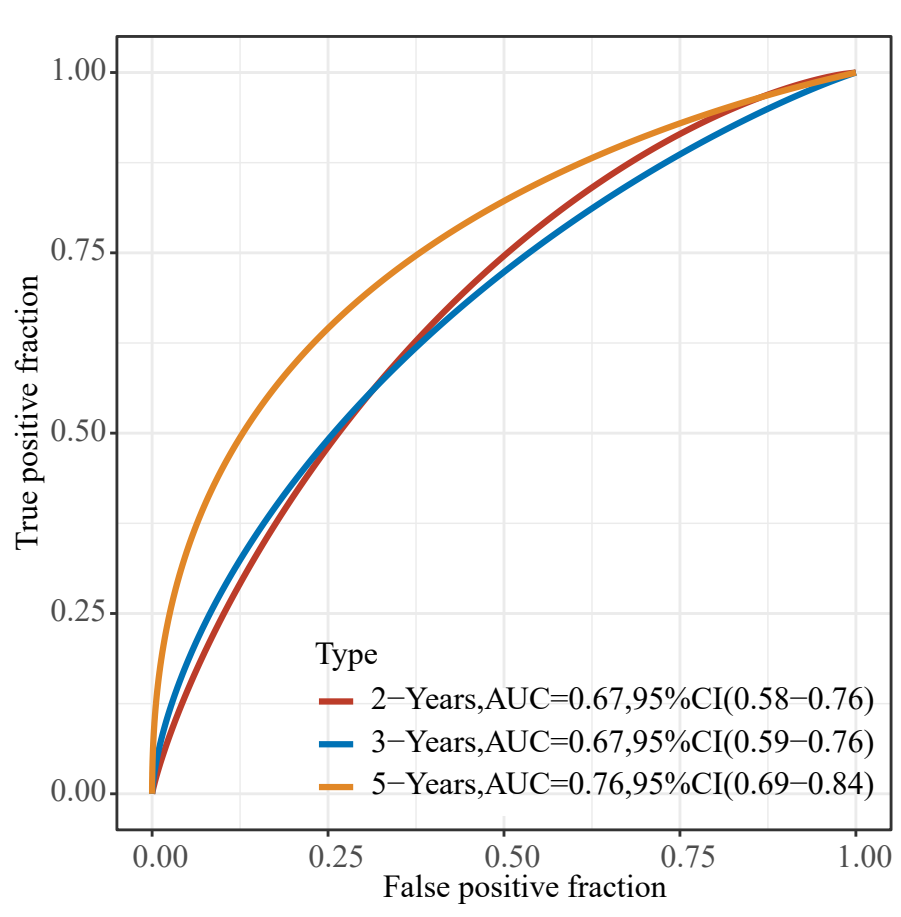

Supplement: Supplementary 2 — Figure S2: clinical prognostic model development and examination. (A) ROC curves and KM survival curves of NPRS in TCGA cohort. (B) ROC curves and KM survival curves of NPRS in GSE103091 queue. (C) ROC curves and KM survival curves of NPRS in cBioPortal queue. [file 8427767.f2.pdf]

t-tests  $p=0.0072$

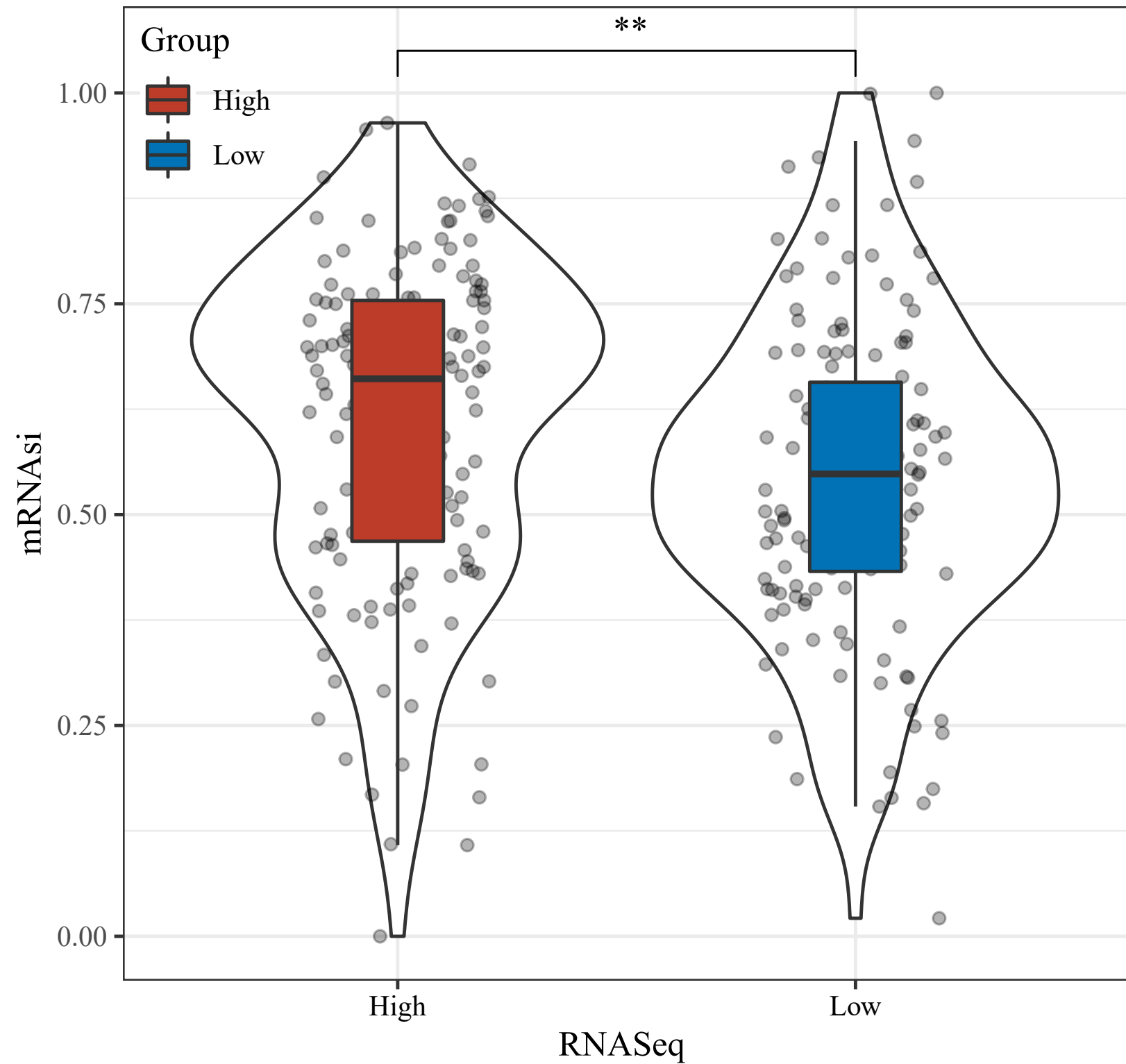

Supplement: Supplementary 3 — Figure S3: high group presented higher mRNAsi relative to low group. [file 8427767.f3.pdf]
